# Hand vs. leg-heel: Evaluating a viable second line approach for chest compressions to bridge the ‘bystander’s window’

**DOI:** 10.1016/j.resplu.2025.100891

**Published:** 2025-02-03

**Authors:** Antje Degel, Shufan Huo, Hans-Christian Mochmann, Jan Breckwoldt

**Affiliations:** a Department of Cardiology, Angiology and Intensive Care Medicine, Deutsches Herzzentrum der Charité, Hindenburgdamm 30 12203 Berlin, Germany; b Dept. of Neurology and Experimental Neurology, Charité Campus Mitte, Charité – University Medicine Berlin, Berlin, Germany; c Department of Neurology, Yale School of Medicine, New Haven, United States; d Dept. of Cardiology and Pulmology, Klinikum Garmisch Partenkirchen, Germany; e Institute of Anesthesiology, University Hospital Zurich, University of Zurich, Zurich, Switzerland

**Keywords:** Cardiopulmonary resuscitation, Chest compressions, Bystander resuscitation, Leg-heel resuscitation, Manikin

## Abstract

**Introduction:**

High quality bystander cardiopulmonary resuscitation (CPR) substantially improves outcomes from cardiac arrest. However, chest compression (CC) quality may be impaired in situations of physical incapacitation, low body weight or rescuer fatigue. For such situations, the leg-heel’-approach has been proposed as an alternative. No study, however, has yet explored this method in a standardized setting over a realistic time span, e.g., until professional rescue teams arrive.

**Methods:**

In a cross-over design, final year medical students performed continuous CC on a manikin using conventional (C-CPR) and ‘leg-heel’-CPR (LH-CPR) for five minutes each with no pause between methods. Students were randomly assigned to the order of approaches. For the LH-CPR, a chair was provided for the rescuer to stabilize the upper body.

**Results:**

121 students were included, and all participants completed the whole ten-minute-task.

Mean absolute CC depth (C-CPR: 49.8 mm [SD 8.7, CI 48.2–51.4] vs. LH-CPR: 49.9 mm [SD 9.4, CI 48.2–51.5], *p* = 0.974) and mean leaning depth (C-CPR: 10.9 mm [SD 7.4, CI 9.6–12.3] vs. LH-CPR: 10.9 [SD 7.6, CI 9.6–12.3]), were similar, while mean CC frequency was higher in C-CPR (120/min [SD 13, CI 118–123] vs. 113/min [SD 16, 110–116], *p* < 0.01). With C-CPR, CC rate steadily increased over time up to 125/min whereas with LH-CPR it remained within the guideline target of 100–120/min. Over time, rescuer fatigue was slightly less pronounced in LH-CPR.

**Discussion:**

In a standardized setting over a realistic time span, the ‘leg-heel’-approach led to equal CPR quality as the conventional approach. Application of the ‘leg-heel’-approach however, has to be considered with caution as its effects on haemodynamics and resuscitation-related injuries are unknown. Cases should therefore be carefully observed.

**Summary:**

This finding may justify developing training algorithms for ‘leg-heel’-CPR as a second line alternative in situations of fatigue, low body weight or physical incapacitation.

## Introduction

Survival and good neurological outcome from out-of-hospital cardiac arrest (OHCA) strongly depend on bystander CPR (BCPR) to bridge the time interval between collapse and the arrival of Emergency Medical Services (the ‘bystanders window’).^1^ Performing high quality chest compressions (CC) is crucial for effective BCPR,^2^, ^3^, ^4^, ^5^ however, BCPR rates remain suboptimal^6^, ^7^ and BCPR quality is highly variable.^8^, ^9^

The quality of BCPR may specifically be limited under the conditions of cardiac arrest in private settings, where more than 70% of OHCA occur.^8^, ^9^, ^10^, ^11^, ^12^ Typically, bystanders in such settings are elderly citizens who initiate BCPR less frequently,^1^, ^13^, ^14^ are more likely to hold physical incapacitations, are single rescuers and are prone to fatigue. In these situations, it is challenging to bridge the time of at least 7 min until EMS arrives on scene.^1^, ^15^ A study on BLS training for elderly volunteers showed that 6% of the participants were unable to kneel and 18% were not capable of performing adequate conventional CC.^15^ Furthermore, even the performance of physically adept bystanders deteriorates after 1–3 min of CC.^16^, ^17^, ^18^

To overcome fatigue and physical limitations, an alternative method for CC was proposed back in 1978 – the foot (‘leg-heel’) approach (LH-CPR).^19^ Despite being recognized in the 2005 ILCOR consensus on science and treatment recommendations,^20^ its level of dissemination and application is unknown. Low-powered historical studies have shown no advantages of LH-CPR over conventional CPR.^21^, ^22^, ^23^ More recent studies compared LH-CPR and conventional CPR in different populations and settings and found equivalent,^24^ superior,^15^ and inferior results^25^ for the ‘leg-heel’ approach. In one study with elderly citizens, female participants performed significantly worse than males in conventional CPR but equally well in LH-CPR.^15^ The authors suggested this relevant target group should be instructed in the LH-CPR as a second-line approach or as an additional option in case of fatigue. However, all the studies were limited by analyzing very short intervention times (2 min per method),^24^, ^25^, ^26^ or by providing resting pauses between the two approaches,^24^, ^25^ or by giving in-time feedback to the participants on CC rate and depth.^15^

In summary, no investigation has yet offered an objective comparison between LH-CPR and conventional manual CPR for the typical conditions of single rescuer BCPR in a private setting to bridge the ‘bystander’s window’. The aim of this study was therefore to compare the CC quality and characteristics of the two methods in an optimized standard setting.

## Methods

### Study design

We conducted a crossover trial for which participants were asked to perform continuous CC on a manikin (ResusciAnne, Laerdal, Norway) using conventional (C-CPR) and ‘leg-heel’-CPR (LH-CPR) for five minutes each. No feedback pertaining CPR quality was given during the study period. Participants were randomly assigned to the order of approaches, and no pause was permitted between methods (to resemble the circumstances of a single-rescuer situation). The total time of 10 min was chosen to mimic the bystander’s window. A brief theoretical explanation of LH-CPR was provided verbally immediately before the start of the CPR sequences. A chair was provided to stabilize the upper body of the participant. The choice of position in relation to the manikin for LH-CPR was left to the participants. To ensure a better haptic experience during the task,^19^ the participants were asked to take off the shoe of the ‘working foot’.

### Ethics approval

Approval from the institutional review board on ethics was obtained (#EA4/225/17) and informed consent was procured. Participation was voluntary, no stipend was awarded, and participation was affirmed to have no influence on any marks given.

### Participants

Participants were final year medical students who had completed an ERC/ILCOR-compliant 5-day emergency medicine course including training in conventional CPR on the day of their participation in the study. ‘Leg-heel’ CPR was not trained during the course and was only verbally explained immediately prior to the trial. We chose advanced medical students at the end of their curricular CPR course as a well-defined ‘gold standard’, fully competent in conventional CC. To attenuate the importance of the experimental ‘leg-heel’ approach, a brief verbal explanation of LH-CPR was only provided during the study itself.

### Setting

Trials were conducted at the Charité Training Centre at Campus Benjamin Franklin, the location of the emergency medicine course. A ‘ResusciAnne’ manikin (Laerdal, Norway) was used for the trial, and performance data was recorded with ‘Laerdal SkillReporter’ (Laerdal, Norway). The participants were familiar with the manikin from the preceding emergency medicine course.

### Data and variables

A questionnaire obtained information on age, gender, weight, height, study semester, previous professional experience defined as previous health care education (e.g., nurse, emergency medical technician, midwife, physiotherapy) and professional work, level of sports activities and physical incapacitations. Data on CC quality was collected with Laerdal SkillReporter. Primary outcome variables included the absolute, effective and leaning depths, as well as the average and instant CC rate. Absolute depth described the overall downward compression of the chest from neutral position, leaning depth described the difference between the neutral position and the remaining chest position at the end of the release phase. Effective depth was computed as difference between absolute and leaning depth, i.e. the factual change in depth between compression and release.

Fatigue was operationalized as decline in compression depth over time. Chest compression fraction was computed and analyzed as surrogate parameter for CPR quality.

### Sample size, randomization, and Statistical analysis

A power analysis with an *α* of 0.10 and a *β* of 0.05 was conducted, yielding a minimum of 114 participants. Participants were allocated to the order of approaches via a computer-aided randomization tool. Statistical analyses were performed with SPSS 19, 23 and 30.0.0 (IBM) and STATA/IC 14.2 (StataCorp. 2015. Stata Statistical Software: Release 14. College Station, TX: StataCorp LP) using descriptive, explorative analyses and Levene and T-Test for significance. To control for potential confounders, we also analyzed the impact of demographic characteristics on CPR performance. Multivariate ANOVAs were used to identify relationships between attributes and CPR performance.

## Results

Overall, 128 students participated in the study. Due to loss of data (theft of laptop), only 121 students were included [43 males, 78 females; age: 26 (SD 3.3, range 21–40); median body weight: 68 kg] (for detailed information see Table 1). 61 students were randomised to group 1 (first C-CPR then LH-CPR) and 60 to group 2 (vice versa). All participants completed the task over the whole ten-minute-period. In LH-CPR, almost all participants placed themselves in the same position to the manikin as in C-CPR, standing beside the thorax on one leg while applying compressions with the heel of the other foot.

**Table 1:** Demographic attributes of participants.

	Female	Male	Overall
Participants *n* (%)	**78** (64.5)	**43** (35.5)	**121** (100)
Age [years] mean (SD, range)	**25.6** (2.9, 21–38)	**26.9** (3.9, 23–40)	**26.0** (3.27, 21–40)
Height [cm] mean (SD, range)	**168.9** (6.5, 153–187)	**183.7** (5.4, 171–194)	**174.1** (9.2, 153–194)
Weight [kg] mean (SD, range)	**62.4** (10.1, 43–102)	**79.3** (7.3, 65–97)	**68.3** (12.1, 43–102)
Sports activities [h/week] mean (SD, range)	**2.7** (2.3, 0–13)	**3.7** (2.3, 0–10)	**3.0** (2.4, 0–13)
Previous professional experience^1^	15/78 (**19.2%**)	6/43 (**14.0%**)	21/121 (**17.4%**)

1 Nurse, emergency medical technician, midwife, physiotherapy.

**Fig. 1:**
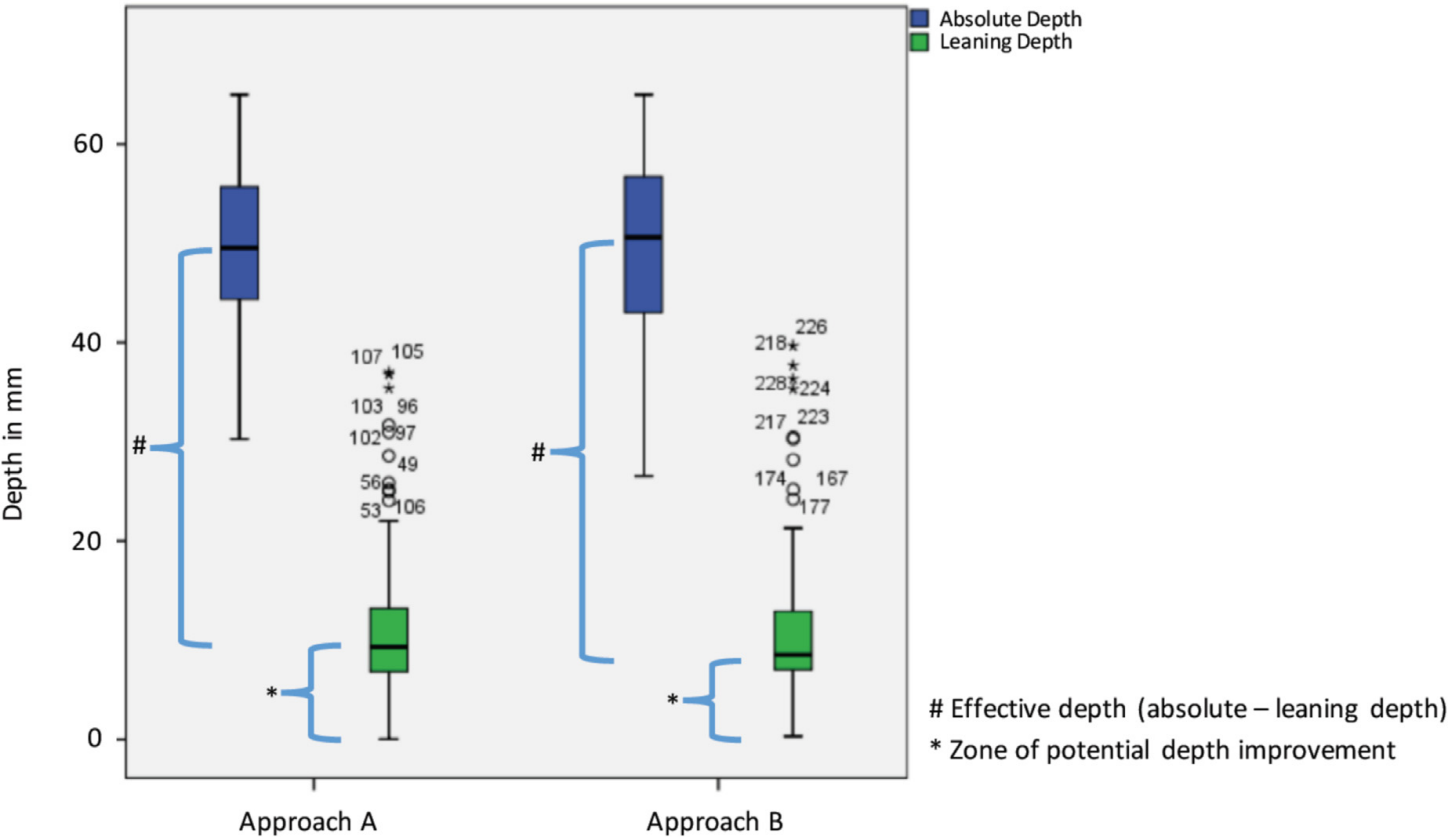
Absolute and leaning depths for conventional CPR and leg-heel CPR with identification of the zone of potential depth improvement.*

Mean absolute CC depth was within the target range for both groups (see Table 2). Mean leaning depth was outside the target for both approaches (Fig. 1). This resulted in a mean effective CC depth outside guideline recommendation (both <40 mm) (see Table 2). Chest compression fraction (CCF) was 99.8% [SD 1.01; CI 99.6–100.0] for C-CPR and 98.8% [SD 2.67, CI 98.3–99.8] for LH-CPR. This difference was statistically significant (*p* < 0.001) with a small effect size (Cohen’s d point estimate 0.34).

**Table 2:** Comparison of compression quality between C-CPR and LH-CPR.

	C-CPR	LH-CPR	Level of significance
Absolute depth [mm]	**49.8** mm [SD 8.7, CI 48.2–51.4]	**49.9** mm [SD 9.4, CI 48.2–51.5]	*p* = 0.953
Leaning depth [mm]	**10.9** mm [SD 7.4, CI 9.6–12.3]	**10.9** mm [SD 7.6, CI 9.6–12.3]	*p* = 0.877
Effective depth [mm]	**38.8** mm [SD 7.9, CI 37.4–40.3]	**38.9** mm [SD 7.9, CI 37.5–40.3]	*p* = 0.871
Compression fraction [%]	**99.8**% [SD 1.01; CI 99.6–100.0]	**98.8** [SD 2.67, CI 98.3–99.8]	*p* < 0.001
CC Frequency	**120** [SD 13, CI 118–123]	**113** [SD 16, 110–116]	*p* < 0.001

Effective CC depth for males was better than for females for both approaches (41.5 [SD 7.4, CI 39.3–43.8] mm and 37.3 [SD 7.9, CI 35.5–39.1] mm respectively, *p* < 0.005 for C-CPR; mean 41 [SD 7.9, CI 38.6–43.5] mm and 37.8 [SD 7.7, CI 36.0–39.5] mm respectively, *p* < 0.028 for LH-CPR). Lower body weight (≤55 kg) led to decreased absolute CC depth in C-CPR (*p* < 0.032) but had no influence in LH-CPR (*p* = 0.123). Weight and height highly correlated with participant sex, and after adjustment for weight and height, the difference in performance was not significant between males and females. CC depth was not influenced by prior experience, age, or physical activity. For both randomization groups, the first approach used led to higher compression depths than the second one.

**Fig. 2:**
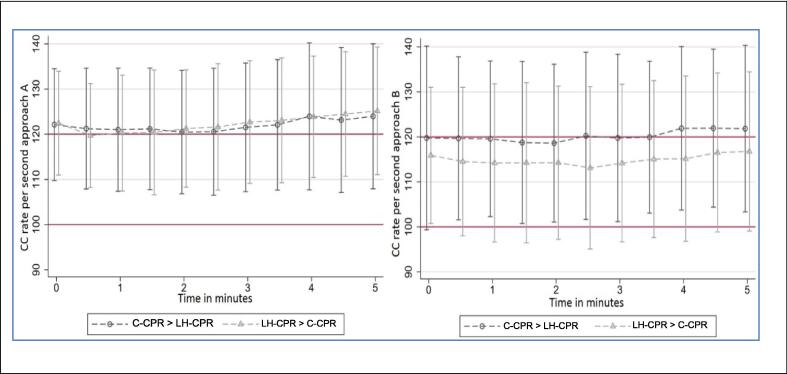
CC rate over time in approach A (conventional CPR; left part of figure) and B (leg-heel CPR; right part of figure). Differentiation by first method, either conventional CPR first (C-CPR > LH-CPR), or leg-heel CPR first (LH-CPR > C-CPR. Red lines indicate the CC target zone (100–120/min). (For interpretation of the references to colour in this figure legend, the reader is referred to the web version of this article.)

Mean CC frequency was within target range for both approaches (see Table 2). With conventional CC, mean compression rate oscillated around 120 /min in the first 2 min and then steadily increased up to 125 /min, whereas with LH-CPR, mean compression rate almost always remained within the target range of 100–120 /min (Fig. 2).

**Fig. 3:**
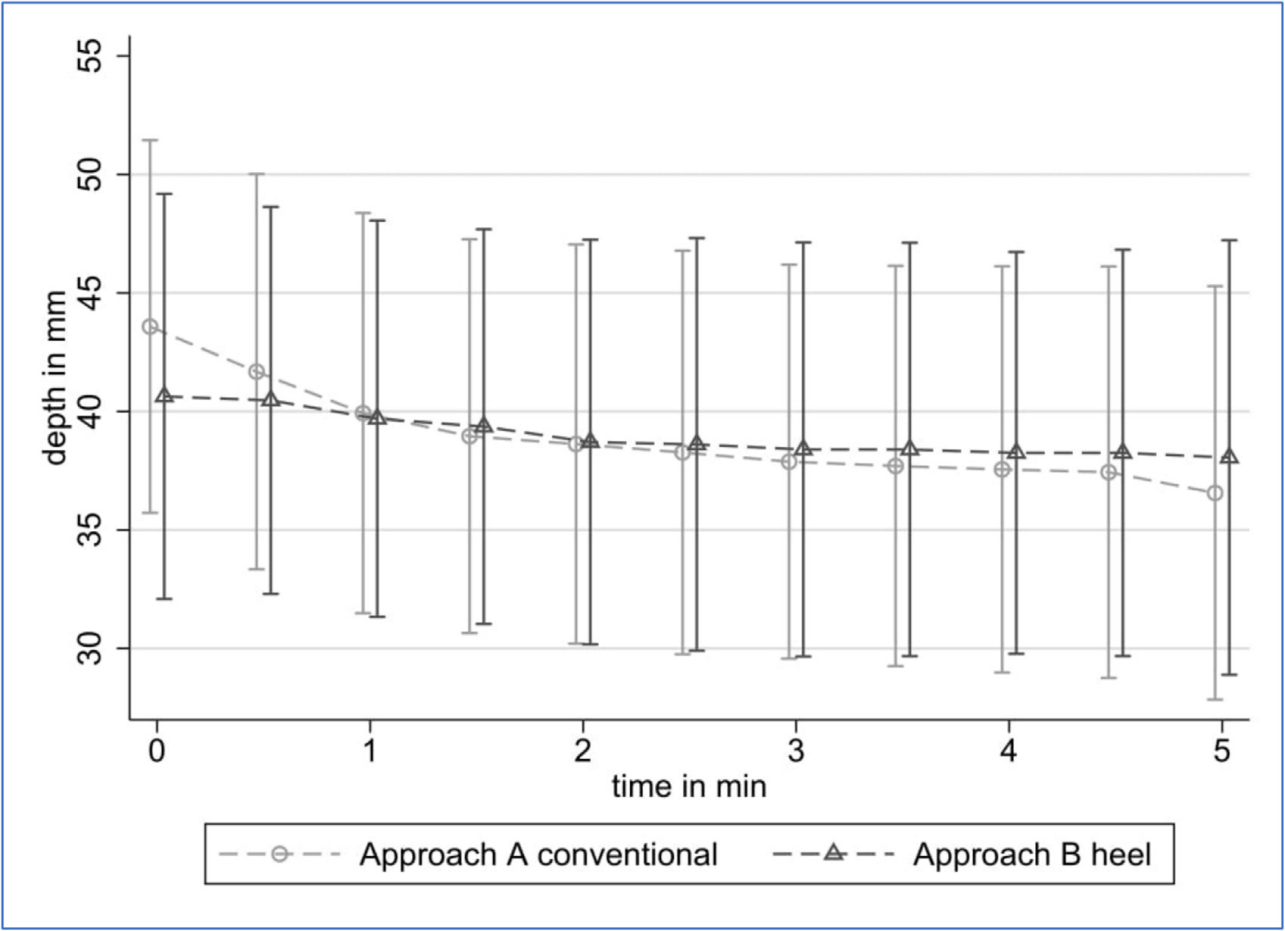
Effective depth of conventional CPR (approach A) and leg-heel CPR (approach B) over time.

**Fig. 4:**
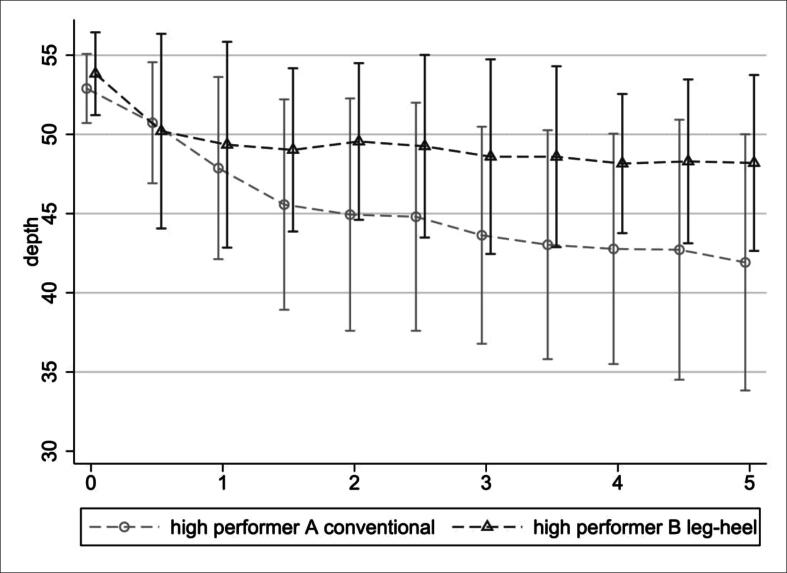
Effective depth of conventional CPR (approach A) and leg-heel CPR (approach B) over time in the subgroup of high performers.

Objectively measured fatigue defined as decline in decline in compression depth over time was less pronounced in LH-CPR (Fig. 3). While C-CPR started with greater depths it soon aligned with LH-CPR. At the end of the study period, C-CPR depths were lower than in LH-CPR, but this effect was not significant. In a comparison of high-performers of both methods (those starting within guideline-target of CC depth ≥50 mm: C-CPR *n* = 31, LH-CPR *n* = 14), fatigue was more pronounced in C-CPR (Fig. 4).

## Discussion

Physical incapacitation and rescuer fatigue are common limitations in achieving high quality CC,^14^, ^16^, ^27^ and even for bystanders in good physical condition, the time until arrival of professional EMS might be too long to maintain high quality CC. Therefore, it is reasonable to consider the ‘leg-heel’ approach as a valid second line alternative.

Our study provides a head-to-head comparison between the two methods in a standardized setting over the entire time span of the ‘bystander’s window’. We showed that the ‘leg-heel’ approach was equal to conventional CC for absolute, effective, and leaning depths, and that both approaches were well within the target ranges for CC frequency and CCF. While differences in CC frequency and CCF were statistically significant in favour of conventional CC, these differences are not clinically relevant. Compared to the results of other studies, we found better performance characteristics for LH-CPR. For the study by Kherbeche et al. a potential explanation might be the different levels of CPR expertise between the medical students in our study, and the school children in Kherbeche’s.^26^ A possible reason for the lower LH-CPR performance in the study by Otero-Agra could be that the participants in that study were explicitly advised to leave their shoes on for the intervention.^25^

Regarding fatigue, our results are difficult to compare to the older studies as these investigated two-rescuer CPR including ventilations. The more recent studies are also difficult to compare as these investigated no more than 2 min of CPR,^24^, ^25^, ^26^ and, in addition, introduced resting phases between the approaches.^24^, ^25^ In our study, we could not show significant differences for fatigue in the overall sample, however, fatigue was less pronounced for LH-CPR in the subgroup of individuals reaching the target depth (‘high performers’). Even more, adequate CC rates were better maintained over time, and compression depth was kept within guideline targets over the whole 10 min with LH-CPR. As rescuer fatigue is a major problem over longer resuscitation times, switching between LH-CPR and C-CPR might be an effective strategy for a single rescuer. This might be an alternative to strategies introducing intentional interruptions of CPR, which might also improve CC quality and perceived fatigue in single-rescuer CPR but at the expense of longer no-flow times and reduced CCF.^28^, ^29^

An important influence on compression depth and recoil is body weight.^30^ In this respect, our study showed that individuals with less body weight (≤55 kg) performed better with LH-CPR. This confirms previous findings suggesting that rescuers with low body weight could profit from switching to LH-CPR.^19^, ^23^

In respect to training, our study demonstrates that for individuals trained in conventional CPR, LH-CPR can be successfully performed without any prior instructions. Similarly, most other studies introduced LH-CPR with very brief explanations, or pictures of the procedure.^21^, ^24^, ^25^, ^26^ During training, CC feedback devices may help participants to reach adequate CC depth, although this hypothesis has also been questioned.^31^, ^32^ In consequence, it would be easy to include LH-CPR into CPR training as a second-line strategy for specific target groups such as senior citizens or relatives of cardiac risk patients.

The application of LH-CPR, however, must be considered with some caution. It remains unclear whether the method improves patient haemodynamics, or leads to more resuscitation-related injuries. In addition, it is unclear whether ethical considerations might inhibit bystanders from using LH-CPR in real life. Furthermore, teaching of an additional CC method may potentially confuse layperson rescuers. This issue that has been raised for the situation when ventilations are included into the training.^21^, ^22^ Despite these unanswered questions, LH-CPR remains preferable to withholding CC altogether, which clearly results in significantly deteriorated outcomes. Cases treated by LH-CPR should be carefully documented and reviewed.

This study also provided useful insights into the potential shortcomings of current CPR training. Some of the medical students failed to achieve adequate CC depth despite considerable teaching efforts explicitly targeting these skills during the course. The participants were in excellent physical condition, without incapacitations, and it therefore seems unlikely that physical strength was their primary limitation. Instead, we identified leaning depth as the main reason why adequate CC depth was not achieved. This phenomenon was already present when participants initiated CC, and it increased over time. Other studies have reported similar findings^33^, ^34^ and measured the negative effects in terms of endotracheal pressure, venous return, myocardial blood flow as well as coronary and cerebral perfusion pressures. This finding underlines the importance of measuring CC quality and identifies a focus for future teaching.^34^, ^35^

### Limitations

Manikin studies have inherent limitations. It is not possible to measure real effects on (cerebral or cardiac) perfusion in humans or to analyse resuscitation injuries. In addition, our findings are based on the abilities of physically fit students with a mean age of 26 years, who are not representative of the general population. Transfer of the results to the putative BCPR bystander group for which LH-CPR might be useful is even more difficult. However, the aim of this study was to derive data in a ‘gold standard’ setting.

## Summary

In a standardized setting over a time span of the entire ‘bystander’s window’, the ‘leg-heel’ approach led to overall equal CPR quality as conventional CPR. Advantages for ‘leg-heel’ CPR could be shown for CC rate that was kept within guideline range over the whole period studied, whereas it increased over time during conventional CPR. In addition, participants with lower body weight were able to achieve better CC depths with the ‘leg-heel’ approach. Training of ‘leg-heel’ CPR may be an option to improve CC quality as a second-line alternative in situations of fatigue, low body weight, or physical incapacitation.

## Ethics

Approval by the Ethical Committee of the Charité – Universitätsmedizin Berlin (# EA4/225/17).

## Consent to publish

All authors read and approved the final manuscript.

## Availability of data and materials

On request.

## Funding/support

None (academic study).

## Authors' contributions

**JB**, **AD** and **HCM** designed the study; **AD** collected and processed raw data, **SH** performed special analyses (performance over time); **AD** and **HCM** interpreted the data, prepared a draft of the manuscript and finalised the manuscript; **JB** contributed to data interpretation, gave intellectual input to different versions of the manuscript and participated in finalising the manuscript.

## CRediT authorship contribution statement

**Antje Degel:** Writing – review & editing, Writing – original draft, Supervision, Project administration, Methodology, Investigation, Data curation, Conceptualization. **Shufan Huo:** Writing – review & editing, Visualization, Software, Methodology, Formal analysis. **Hans-Christian Mochmann:** Writing – review & editing, Software, Investigation, Data curation, Conceptualization. **Jan Breckwoldt:** Writing – review & editing, Supervision, Methodology, Investigation, Conceptualization.

## Declaration of competing interest

The authors declare the following financial interests/personal relationships which may be considered as potential competing interests: ‘**AD, SH, HCM**: No competing interests to declare. **JB**: Member of the ILCOR EIT Task Force (Education, Implementation and Teams); member of the ERC Science and Education Committee /SEC IES; member of the writing group for the ERC Guidelines 2025 (Chapter Education).’.
